# Efficacy of High-Dose Statins in Preventing Contrast-Induced Nephropathy Post-cardiac Catheterization

**DOI:** 10.7759/cureus.81795

**Published:** 2025-04-06

**Authors:** Montaser Elkholy, Mohammad Akkawi, George G Kidess, Hisham Alsharif, Mohamed Jimale, Ali R Khan, Yasemin Bahar, Wael Aljaroudi, Islam Elgendy, M. Chadi Alraies

**Affiliations:** 1 Department of Internal Medicine, Detroit Medical Center/Sinai Grace Hospital/Wayne State University, Detroit, USA; 2 Department of Internal Medicine, Wayne State University School of Medicine, Detroit, USA; 3 Department of Medicine, Wayne State University School of Medicine, Detroit, USA; 4 Department of Internal Medicine, Ohio State University, Columbus, USA; 5 Department of Internal Medicine, University of Texas Medical Branch at Galveston, Galveston, USA; 6 Department of Cardiology, Wellstar Medical College of Georgia Health, Augusta, USA; 7 Division of Cardiovascular Medicine, Gill Heart Institute, University of Kentucky, Lexington, USA; 8 Department of Cardiology, Wayne State University, Detroit Medical Center, Detroit, USA

**Keywords:** chronic statin therapy, contrast-induced nephropathy, high-dose statins, outcome, percutaneous coronary intervention

## Abstract

Contrast-induced nephropathy (CIN) is defined as an increase in serum creatinine (s-CR) of at least 0.5 mg/dL or a 25% or greater increase from baseline within 48-72 hours of contrast agent administration. High-dose statins have been proposed as a prophylactic measure against CIN. This updated systematic review and meta-analysis aimed to assess the efficacy of peri-procedurally administered high-dose statins in preventing CIN in patients who underwent cardiac catheterization. A comprehensive literature review of PubMed and Embase was conducted with a distinct focus on studies published in the last 15 years up to June 2024. The study included randomized controlled trials (RCTs) that assessed the impact of high-dose statin treatment during or around the time of cardiac catheterization on the rate of contrast-induced nephropathy (CIN). It excluded trials comparing low-dose statins or statins used in non-cardiac surgeries, as well as studies with missing data or unclear end points on CIN. The primary objective was to evaluate the efficacy of high-dose statins administered peri-angiography to prevent CIN. Random effects were used using the DerSimonian and Laird method. A subgroup analysis was performed to assess the effectiveness of high-dose statins in preventing CIN in patients on chronic statin therapy. Our pooled analysis of 2,312 participants revealed a significantly lower incidence of CIN (odds ratio (OR): 0.47, 95% confidence interval (CI): 0.30-0.72, P = 0.0007, I^2^: 38%) in the high-dose statin group compared to the control group. However, the subgroup analysis showed no benefit of high-dose statins in decreasing the incidence of CIN in individuals already on chronic statin therapy (OR: 1.03, 95% CI: 0.33-3.18, P = 0.97). Our study highlights the beneficial effect of high-dose statins in preventing CIN in statin-naive patients. However, no benefit was observed in patients who were on chronic statin therapy.

## Introduction and background

Contrast-induced nephropathy (CIN) is one of the most frequent complications of cardiac catheterization, characterized by acute renal function impairment following exposure to contrast agents. CIN is clinically defined as an increase in serum creatinine (s-CR) of at least 0.5 mg/dL or 25% or more from baseline within 48-72 hours of contrast agent administration. CIN can be associated with increased short-term mortality, longer hospital stays, and increased levels of healthcare costs. The incidence of CIN is estimated to be 0.6%-2.3% in the general patient population [1]. However, higher incidences have been reported in patients with risk factors (up to 20%), including baseline renal dysfunction or other comorbidities [2]. The presence of chronic kidney disease (CKD) is the most substantial predictive factor for developing CIN, with the risk rising as renal function declines. A large-scale observational study of 985,737 patients undergoing percutaneous coronary intervention demonstrated that the incidence of CIN was significantly higher in patients with severe CKD (eGFR < 30 mL/minute/1.73 m^2^) compared to those with normal kidney function at baseline (26.6% versus 5.2%, respectively). Furthermore, acute kidney injury (AKI) secondary to CIN was associated with worse in-hospital outcomes, including in-hospital mortality (9.7% in AKI patients and 34% in patients requiring dialysis versus 0.5% in non-AKI patients) and elevated in-hospital rates of significant bleeding and myocardial infarction [3]. Consequently, identifying feasible strategies for preventing CIN has emerged as a primary focus in clinical research.

Numerous mechanisms have been investigated to explain the pathophysiology of contrast-induced kidney injury. Contrast agents exert a direct cytotoxic effect on renal tubular cells. The pathogenesis of CIN also develops through indirect processes leading to disruptions of renal hemodynamics and excessive production of reactive oxygen species. The renal hemodynamic decline stems from an imbalance between renal vasodilators, such as nitric oxide, and renal vasoconstrictors, including prostaglandins and endothelin. Renal microcirculation impairment occurs, eventually leading to worsened kidney ischemic injuries and increased oxidative stress within the organs [4,5].

The nephroprotective effect of statins has been explored in various studies over the years, with growing interest in their potential role in preventing CIN. They demonstrate pleiotropic benefits that might contribute to renal protection through anti-inflammatory, antioxidant, and endothelial-protective properties [6,7]. One proposed mechanism involves inhibiting isoprenoid synthesis, which reduces oxidative stress and inflammation while promoting nitric oxide synthesis [8]. Given these potential benefits, statins have gained interest among researchers, primarily for patients undergoing procedures involving contrast media.

The evidence on the effectiveness of statins in reducing CIN remains inconclusive [9,10]. In one of the earliest meta-analyses on this topic conducted by Zhang et al., statins did not yield a significant protective effect against CIN [11]. In contrast, Thompson et al. conducted a large meta-analysis that included 7,161 patients and demonstrated a remarkable reduction in the incidence of CIN in statin groups. However, the author acknowledged several limitations, including the considerable heterogeneity among trials and the underrepresentation of patients with chronic kidney disease (eGFR < 60 mL/minute/1.73 m^2^), the population at the highest risk of developing CIN [12].

This updated systematic review and meta-analysis aimed to determine how efficiently high-dose statins prevent CIN in patients undergoing heart catheterization. We intend to analyze data from randomized controlled trials (RCTs) to further understand the extent to which high-dose statins can reduce the risk of CIN.

We also aimed to investigate whether the protective effects of high-dose statins differ in patients with prior statin use history, given a significant proportion of individuals are already on long-term statin therapy. ​The impact of high-dose statin therapy on the incidence of contrast-induced nephropathy (CIN) in patients on chronic statin therapy has not been adequately investigated. One small single-center trial found no benefit of atorvastatin reloading in CIN prevention [13]. However, this aspect has been overlooked in previous meta-analyses, making our study particularly relevant to clinical practice. Our analysis extends prior reviews by employing PubMed search up to June 2024 and employing more liberal RCT eligibility criteria. This meta-analysis only incorporated RCTs because they remain the gold standard as they help minimize bias, establish causality, and ensure fair comparisons [14]. We anticipate that this comprehensive review will clarify the use and utility of high-dose statins in preventing CIN and help clinicians make more informed decisions for patients undergoing cardiac catheterization.

## Review

Methods

Study Design and Data Sources

The current study is a systematic review and meta-analysis performed based on the guidelines of the Preferred Reporting Items for Systematic Reviews and Meta-Analyses (PRISMA) statement [15]. The primary objective of this review was to assess the effectiveness of high-intensity statin therapy for the prevention of contrast-induced nephropathy in patients who are to undergo cardiac catheterization. Two independent researchers performed a comprehensive literature search in PubMed and Embase databases, aiming at covering the studies published from January 2009 to June 2024. We searched for grey literature, including theses, reports, and other non-peer-reviewed sources. Additionally, we reviewed conference proceedings and preprints from relevant databases to identify studies not published in peer-reviewed journals. The search was structured using keywords related to the subject: high-dose statins, contrast-induced nephropathy, cardiac catheterization, and prevention. A search approach was used employing keywords and MeSH terms (Appendices). Studies that provided information on the incidence of CIN or short-term mortality after administration of high-dose statin around the time of angiography were included. Only articles published in English were included in this meta-analysis to guarantee uniformity and standardization in study interpretation due to resource limitations and the level of expertise needed for appropriate translation.

Eligibility Criteria

We included randomized controlled trials (RCTs) that focused on distinguishing the impact of high-dose statin treatment at the time of cardiac catheterization or shortly before or after that on the rate of CIN. Randomized trials that only included comparisons of low-dose statins or statins used during non-cardiac-related operations were excluded. Furthermore, studies with missing data or lacking well-defined end points on CIN were also excluded. We included adult patients undergoing elective or urgent coronary angiography without sample size or age limit and without any specific comorbidity.

Data Extraction and Quality Assessment

Study characteristics, patient demographics, statin regimen, including dose and timing of the statin, and outcomes were extracted by two independent reviewers (ME and MA). In cases where two reviewers disagreed, the discrepancy was discussed until a consensus was reached, or a third reviewer was consulted. The baseline characteristics are included in the Appendices. The primary end point assessed was acute kidney injury (AKI)-CIN, defined as an absolute increase in serum creatinine by ≥0.5 mg/dL or ≥25% of baseline within 48-72 hours following the procedure. To assess the risk of bias in the incorporated trials, we used the Cochrane Risk of Bias tool for RCTs [16].

Subgroup Analysis

Subgroup analysis was done to determine the effect of high-dose statin in patients who were already on chronic statin treatment. This study aimed to determine whether high-dose statins can prevent CIN better in statin-naive versus ongoing statin users prior to cardiac catheterization.

Statistical and Sensitivity Analysis

Data were synthesized using the Cochrane Collaboration RevMan Web. For dichotomous data, random effects were done using the DerSimonian and Laird method, with results expressed in odds ratio (OR) and 95% confidence intervals (CIs). Inter-study heterogeneity was estimated by an I² threshold of 25%-50% (mild), 50%-75% (moderate), and >75% (significant). An appropriate approach to pooling was used through the random effects model. The results were tested for robustness by performing sensitivity analyses wherein studies with a high risk of bias were removed.

Results

**Figure 1:**
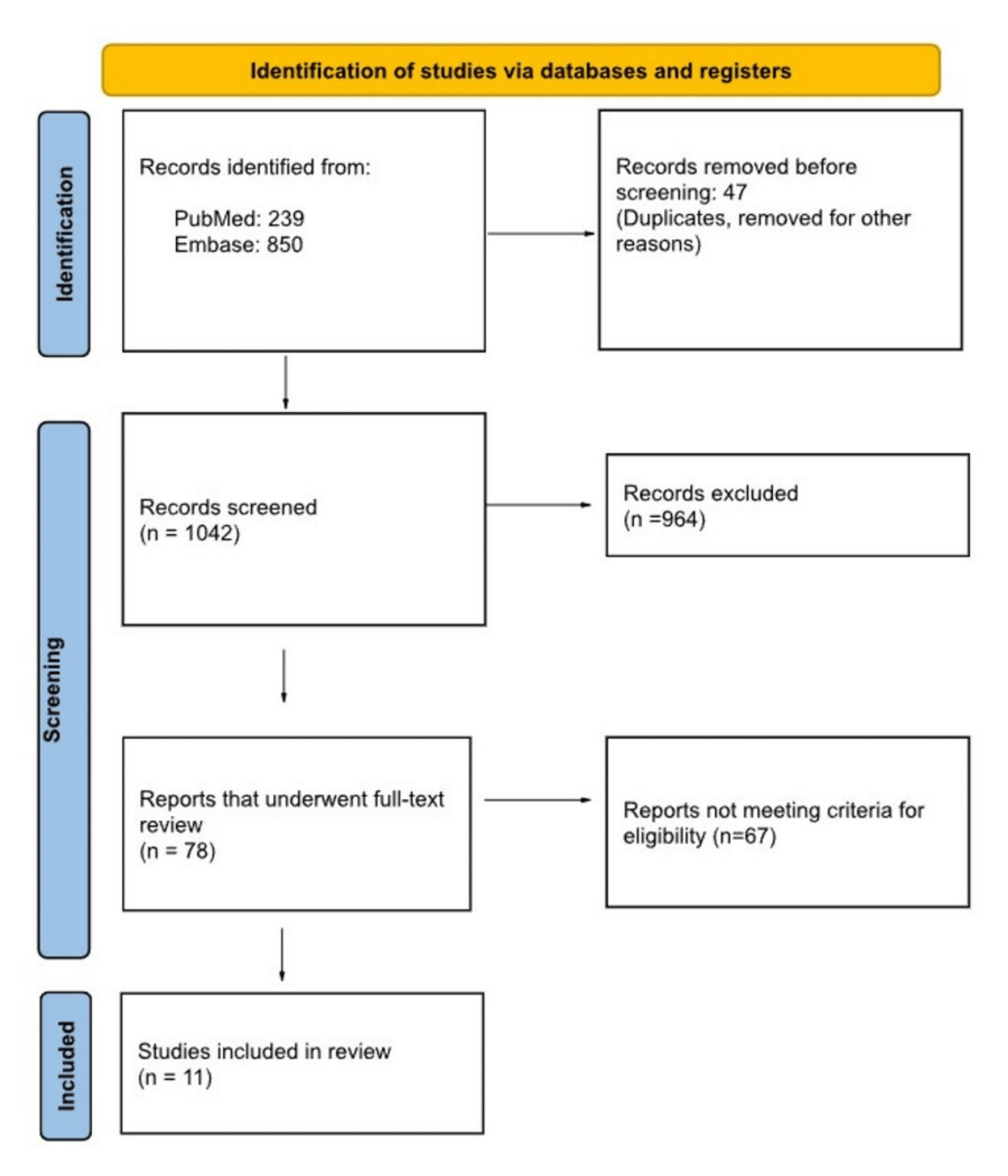
PRISMA Flow Diagram Illustrating the Identification, Screening, Selection, and Inclusion of Studies for Final Analysis PRISMA: Preferred Reporting Items for Systematic Reviews and Meta-Analyses

Our systematic literature search yielded an initial pool of 1,089 potential articles. Following the removal of 47 duplicate records, a total of 1,042 articles underwent title and abstract screening. This initial screening process resulted in the selection of 78 articles for full-text review. After careful evaluation, we included 11 randomized controlled trials that met our predefined eligibility criteria (Figure 1) [13,17-26].

The details of the included studies are given in Table 1 with regard to sample size, dosage frequency, and efficacy measures.

**Table 1:** Characteristics of the Included Studies CIN: contrast-induced nephropathy, RCT: randomized controlled trial, ACS: acute coronary syndrome, STEMI: ST-elevation myocardial infarction, NAC: N-acetyl cysteine, CI-AKI: contrast-induced acute kidney injury, CAD: coronary artery disease, PCI: percutaneous coronary intervention, GFR: glomerular filtration rate, NSTEMI: non-ST-elevation myocardial infarction, CKD: chronic kidney disease, eGFR: estimated glomerular filtration rate, DM: diabetes mellitus

Study	Country	Design	Inclusion criteria	CIN definition	Intervention	Statin dose	Comparison	Intervention sample size	Comparison sample size	Key findings
Leoncini et al. (2014) [17]	Italy	Single-center double-blinded RCT	Patients naive to statins with ACS without STEMI	Increase in serum creatinine of greater than or equal to 0.5 mg/dL or greater than or equal to 25% increase over the baseline value within 72 hours of contrast agent administration	Rosuvastatin + isotonic saline + NAC	40 mg on admission and 20 mg/day until procedure (continued upon discharge)	Isotonic saline + NAC	252	252	High-dose rosuvastatin can prevent CI-AKI and improve short-term clinical outcomes.
Abaci et al. (2015) [18]	Türkiye	Single-center RCT	Patients naive to statins and scheduled for elective coronary angiography	Increase in serum creatinine of 0.5 mg/dL or an absolute increase of 25% from baseline <48 or 72 hours after contrast exposure	Rosuvastatin + isotonic saline	40 mg <24 hours before the procedure and then 20 mg/day for 2 days after the procedure	Isotonic saline	103	105	Statins reduced the incidence of CIN.
de Oliveira et al. (2012) [19]	Brazil	Single-center open-label RCT	Patients with CAD referred for elective PCI	Absolute increase in serum creatinine level to ≥0.5 mg/dL or a ≥25% increase in the creatinine level in 24 hours	Rosuvastatin	40 mg administered 2-6 hours before PCI	No high-dose rosuvastatin treatment before PCI	67	68	The use of a preloaded dose of rosuvastatin did not have a protective effect on renal function in chronic statin users undergoing elective PCI.
Bidram et al. (2015) [20]	Iran	Single-center double-blinded RCT	Patients with chronic stable angina who were referred for coronary angiography and did not undergo PCI	Increase in post-procedural serum creatinine of >0.5 mg/dL or >25% from baseline in the absence of any other causes	Atorvastatin + isotonic saline	80 mg 12 hours before contrast injection	Isotonic saline + placebo	100	100	Pre-operation short-term high-dose atorvastatin use was associated with a significant decrease in serum creatinine level and an increase in GFR after angiography.
Patti et al. (2011) [21]	Italy	Multi-center double-blinded RCT	Statin-naive patients with NSTEMI or unstable angina and a planned invasive strategy within 48 hours	Post-intervention increase in serum creatinine of >0.5 mg/dL or >25% from baseline	Atorvastatin + isotonic saline	80 mg loading dose 12 hours before coronary angiography with another 40 mg dose 2 hours before the procedure	Isotonic saline + placebo 12 hours before angiography, further placebo dose 2 hours before	120	121	Short-term pretreatment with high-dose atorvastatin load prevents CIN and shortens hospital stay in patients with ACS undergoing PCI
Quintavalle et al. (2012) [22]	Italy	Multi-center RCT	Statin-naive patients with CKD scheduled for elective coronary angiography due to symptomatic CAD or PCI in de novo lesions in native coronary arteries	Increase in serum cystatin C and serum creatinine concentration 10% above the baseline value at 24 hours after the administration of contrast	Atorvastatin + NAC + sodium bicarbonate	80 mg loading dose within 24 hours before contrast administration	NAC + sodium bicarbonate	202	208	A single high-loading dose of atorvastatin administered within 24 hours before contrast exposure is effective in reducing the rate of CIN.
Syed et al. (2017) [23]	India	Single-center double-blinded RCT	Serum creatinine between 1 and 1.5 mg/dL or eGFR > 60 mL/minute/1.73 m^2^ and suffering from controlled DM or hypertension	Increase in serum creatinine concentration of 0.5 mg/dL or 25% above the baseline within 48 hours after contrast administration	Atorvastatin + NAC + isotonic saline	80 mg every day from 3 days prior to contrast-medium administration to 2 days following it	NAC + isotonic saline	80	80	The study shows the positive role of statins in preventive strategy against CIN along with NAC.
Khosravi et al. (2016) [24]	Iran	Single-center double-blinded RCT	Patients with diabetes mellitus or CKD (15 < GFR < 60 mL/minute, Cr > 1.5 mg/dL) and an age range of 55-75 years candidate for angiography	Increase in serum creatinine more than 0.5 mg/dL or more than 25% from the baseline values after angiography	Atorvastatin + NAC + isotonic saline	80 mg from 48 hours before angiography	NAC + isotonic saline	110	110	Standard hydration, and NAC and atorvastatin (80 mg) reduced the incidence of CIN.
Shehata et al. (2015) [25]	Egypt	Multi-center double-blinded RCT	Diabetic patients, carrying the diagnosis of chronic stable angina and suffering from mild or moderate CKD	Absolute increase of at least 0.5 mg/dL (44.2 μmol/L) or a relative increase of at least 25% in serum creatinine recorded after PCI, in comparison with baseline value	Atorvastatin + NAC + isotonic saline	80 mg daily for 2 days before angiography	Placebo + NAC + isotonic saline	65	65	Atorvastatin dose of 80 mg per day for 48 hours is associated with decreased incidence of CIN in diabetic patients with CKD undergoing PCI.
Hammami et al. (2023) [13]	Tunisia	Single-center single-blinded RCT	All patients undergoing coronary angiography or PCI	Increase in serum cystatin C concentration by 10% above the baseline value 24 hours after contrast media administration or the incidence of serum creatinine-based CIN defined as the increase in serum creatinine concentration of 44.2 mmol/L or 25% above baseline within 72 hours after exposure to contrast media	Atorvastatin + normal saline	Loading a high-dose 80 mg of atorvastatin 1 day before and 3 days after the coronary procedure	Received usual dose of atorvastatin 10/20/40 mg + normal saline	56	54	The study did not find a benefit of systematic atorvastatin reloading in patients on chronic atorvastatin therapy in preventing CIN.
Watanabe et al. (2022) [26]	Japan	Multi-center open-label RCT	Patients with CKD (eGFR < 60 mL/minute/1.73m^2^ or proteinuria) scheduled for cardiovascular catheterization and intervention such as coronary angiography, PCI, catheter ablation, or endovascular treatment	Increase in serum creatinine of 0.5 mg/dL or 25% above baseline at 48 hours after contrast medium exposure	Pitavastatin + isotonic saline + sodium bicarbonate	High-dose pitavastatin (4 mg/day × 4 days) on the day before the procedure and 2 days after the procedure	Isotonic saline + sodium bicarbonate	203	203	High-dose pitavastatin increased the incidence of CIN in this study population.

Outcome: Incidence of Contrast-Induced Nephropathy

**Figure 2:**
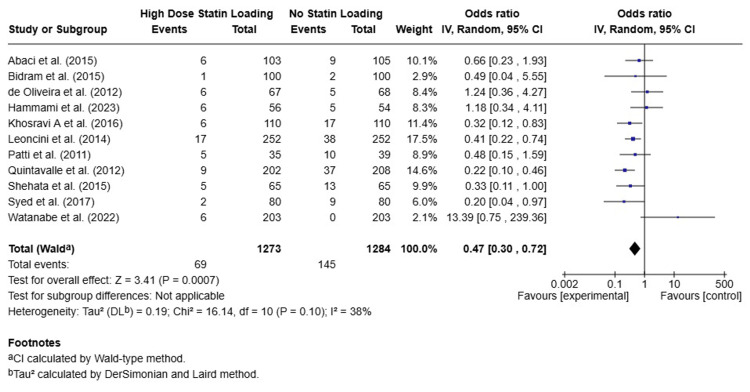
Forest Plot of Contrast-Induced Nephropathy Incidence in High-Dose Statins Versus Control

The pooled analysis showed that high-dose statins significantly reduced the incidence of CIN compared to the control group (OR: 0.47, 95% CI: 0.30-0.72, P = 0.0007). This effect was consistent across studies, and the overall heterogeneity was mild (I² = 38%). A forest plot of the individual study results for CIN incidence is presented in Figure 2.

The pooled absolute risk reduction (ARR) is 5.87%, and the number needed to treat is 17.

Subgroup Analysis

**Figure 3:**
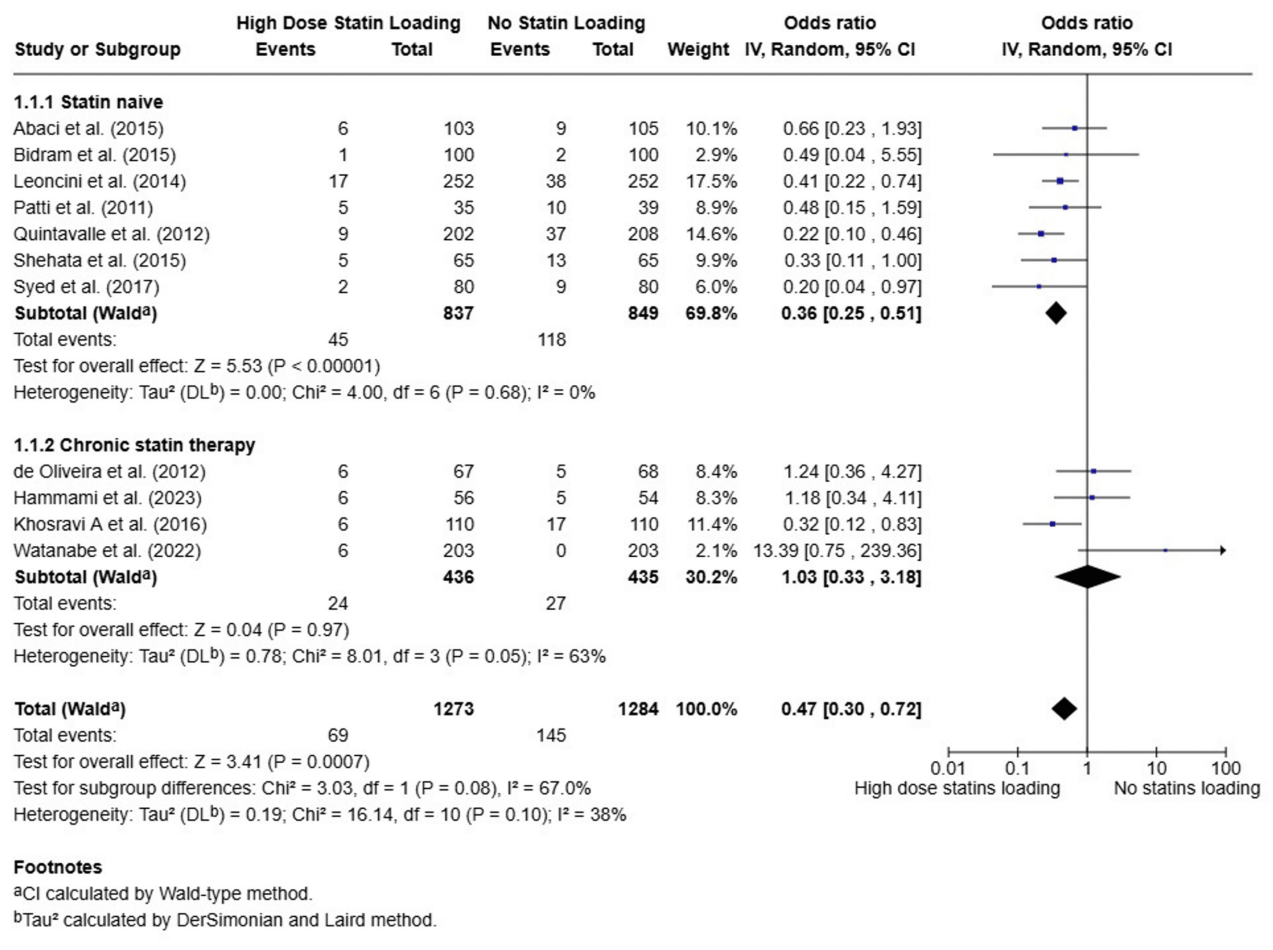
Forest Plot of Subgroup Analysis of the Effect of High-Dose Statins on Contrast-Induced Nephropathy Incidence in Patients Already on Chronic Statin Therapy

A subgroup analysis was performed to assess whether the efficacy of high-dose statins varied between patients who had not previously taken statins and patients who had. Among statin-naive patients, high-dose statins significantly reduced the incidence of CIN (OR: 0.36, 95% CI: 0.25-0.51, P < 0.0001). However, in patients on chronic statin therapy prior to the procedure, high-dose statins did not show any significant benefit in reducing CIN (OR: 1.03, 95% CI: 0.33-3.18, P = 0.97) (Figure 3).

Sensitivity Analysis

The sensitivity analysis results revealed that the total estimate of the primary outcome was stable. Publication bias was not found to have a strong influence on the compiled odds ratio because when studies with a high risk of bias, together with small sample sizes, were excluded, the calculated odds ratio did not change (Appendices). In addition, the analysis of sensitivity, whereby the significance of the effect was tested in turn excluding one study each time, revealed that no individual study had a dominant impact.

Adverse Events

The overall incidence of high-dose statin side effects such as myopathy or asymptomatic increase in liver enzymes was observed in one or a few trials only. The risk of any adverse event was comparable in the high-dose statin and the control groups without much variation.

Discussion

Contrast-induced nephropathy is a common complication of percutaneous coronary intervention (PCI) that is associated with an increased risk of cardiovascular complications and mortality [27]. Some studies have shown that factors such as increased age, elevated serum creatinine, and hypotension might increase the risk for CIN [4]. It is also essential to highlight that social disparities in preventive care and inequities in cardiovascular therapies and baseline comorbidities can also influence CIN risk disproportionally based on race or socioeconomic status [28]. Several mechanisms have been proposed, including intrarenal vasoconstriction, oxidative stress, and increased inflammation, all of which could lead to impaired renal function [29]. Given the variety of these mechanisms, many preventive methods have been proposed, such as pre-procedural hydration, limited contrast use, and medications such as N-acetyl cysteine, vitamin C, and adenosine antagonists [27,30]. Pre-procedure statin administration has also been shown in several studies to be effective in reducing the risk of CIN in patients undergoing cardiac catheterization or PCI [30,31]. While the protective effects of statins on CIN have been well-established, data on its effectiveness for patients who are on chronic statin therapy or those who are statin-naive has been limited. This meta-analysis pooled data from multiple RCTs to explore the efficacy of high-dose statin use on CIN while additionally analyzing their effects on patients who are either statin-naive or undergoing chronic statin therapy.

Our study has shown significantly lower odds of CIN in patients treated with high-dose statins compared to those who have not received statins prior to coronary angiography (pooled OR: 0.47, 95% CI: 0.30-0.72, P = 0.0007). This agrees with most previous studies, including the results of several previously performed meta-analyses [30-33]. Although this has been explored in some meta-analyses and a network meta-analysis, to our knowledge, this study is the largest meta-analysis directly comparing randomized controlled trials focusing specifically on the effect of high-dose statins on CIN. While the lipid-lowering effects of statins have been established, several studies have shown that their effects can be pleiotropic, such as by affecting inflammatory pathways and beta-adrenergic signaling, and this has been proposed as a potential mechanism for their nephroprotective effects [9,34]. Interestingly, a study employing path analysis modeling to analyze the effect of statins on CIN has shown that neither their lipid-lowering effect nor their anti-inflammatory effects account for their protective benefits, with another study suggesting that their nephroprotective effect can be better explained by the inhibition of renal tubular apoptosis [31,35]. Contrast media appears to inhibit mitochondrial enzyme activity that results in the release of reactive oxidative species (ROS) that induce apoptosis, which is inhibited by statins in a dose-dependent manner [31,36]. Several previous studies have also shown that treatment with specifically high-dose statins is more effective in the prevention of CIN, including in patients with chronic kidney disease, and that the nephroprotective effect was observed regardless of baseline eGFR [9,32,33].

Additionally, upon subgroup analysis, our study finds that high-dose statin administration significantly reduced the odds of CIN in patients who were statin-naive, but that effect was not observed for those who were on chronic statin therapy. Various studies have shown the benefit of short-term statin therapy for statin-naive patients undergoing coronary angiography to prevent CIN, including high-risk patients [23,37,38]. With regard to patients on chronic statin therapy, our study showed no significant impact of high-dose statins on the incidence of CIN, which can be explained by the propensity of this group to develop rhabdomyolysis. While controversial, some studies have found a higher risk of acute renal injury in patients treated with high-dose statins while on chronic statin therapy [39,40]. Although future research is needed to confirm these findings and delineate specific etiologies, the results of our study showing no benefit of high-dose statins for patients treated with chronic statins with the presumed risk of renal injury found in previous studies might make it reasonable to employ caution when prescribing high statins in this patient population. Researchers have also found that statins inhibit the synthesis of coenzyme Q10 [41,42]. The 28-day placebo-controlled coenzyme Q10 supplementation studies of animals and humans with end-stage renal disease have noted improvements in renal function [42,43]. This was further corroborated in the study by Chen et al., which found that the administration of coenzyme Q10 prior to cardiac catheterization significantly reduced the rate of developing CIN [44]. Previous literature suggests that the potential reasons for this protective effect include neutralization of oxidative damage, prevention of hemodynamic changes, or alleviation of the progression of histologic damage associated with CIN [45]. Perhaps the chronic inhibition of endogenous coenzyme Q10 by chronic statins could limit its nephroprotective effects as seen in our study.

Limitations

As with any meta-analysis and systematic review, a potential limitation of our study is the carry-over of individual study limitations. Additionally, our study did not pool results such as rate of mortality or major adverse cardiovascular events since only three of the 11 pooled studies included that data, which could have provided insights into the impact of high-dose statin on these events. Another potential for improvement could have been subgroup analysis for patients with diabetes, which is an important risk factor for CIN, with some studies suggesting that high-dose statins might be especially helpful in preventing CIN in this patient population [46]. There was also some variability in our included studies with respect to the type of statin administered among the patients, which could have led to variability in outcomes. We also did not account for concurrent medication use, which could have interacted with the high-dose statins and significantly affected outcomes. An inherent limitation of meta-analyses is that we could not include individual patient data, which could have limited our analysis.

Strengths

Despite the mentioned limitations, we believe that our study provides valuable insights. In addition to confirming the impact of high-dose statins on CIN by pooling the results of recently published trials, our study is also one of few that shows a disparity in the effectiveness of high-dose statins depending on prior chronic statin exposure. While future research is needed to provide further evidence for this finding, this can potentially impact clinical decision-making for the pretreatment of patients who are on chronic statin therapy prior to coronary angiography or PCI and suggests that caution prior to prescribing high-dose statin therapy for this patient population might be warranted. To our knowledge, our study is also the most robust meta-analysis to date exploring the effect of high-dose statins on CIN. Our results also show low heterogeneity (I2 = 38%), which increases confidence in our pooled data. Given that inflammation could underlie the pathophysiology of CIN and that high-dose statin therapy has been shown to be useful in preventing CIN for patients who are statin-naïve with high inflammatory markers [47], perhaps it would be useful in future studies to explore the effect of high-dose statin therapy on CIN for patients on chronic statin therapy based on their baseline inflammatory marker levels.

## Conclusions

This meta-analysis highlights that giving high doses of statins to patients undergoing cardiac catheterization lowers the risk of CIN. High-dose statin treatment offers its greatest protective impact to patients who have never taken statins before cardiac catheterization but provides no significant effect to patients on chronic statin therapy. According to these results, patients who are statin-naive may benefit from using high-dose statins as a preventative measure against CIN. However, the lack of benefit in chronic statin users points to the need for further research to understand the underlying mechanisms and explore better prevention strategies. Further studies are needed to verify these findings, particularly among specific patient subgroups.

## Figures and Tables

**Figure 4:**
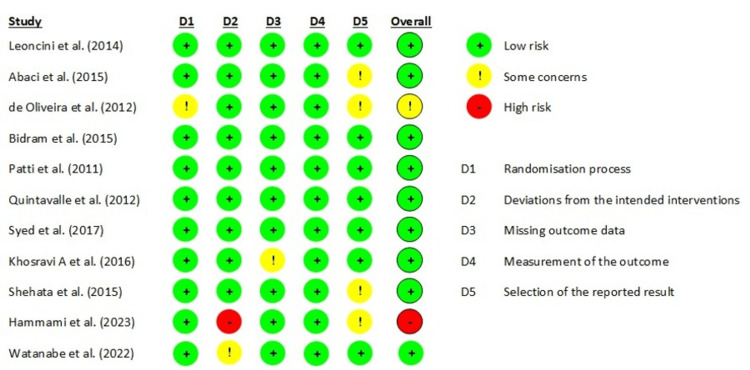
Cochrane RoB Tool Assessment for the Included RCTs Leoncini et al. [17], Abaci et al. [18], de Oliveira et al. [19], Bidram et al. [20], Patti et al. [21], Quintavalle et al. [22], Syed et al. [23], Khosravi et al. [24], Shehata et al. [25], Hammami et al. [13], Watanabe et al. [26] RoB: Risk of Bias, RCTs: randomized controlled trials

## Appendices

The keywords and MeSH terms used in this study are shown in Table 2. 

**Table 2:** Keywords and MeSH Terms

Population	Intervention	Outcome
Coronary angiography Angiography, Coronary Angiographies, Coronary Coronary Angiographies Angioplasty, Coronary Balloon Angioplasties, Coronary Balloon Balloon Angioplasties, Coronary Balloon Angioplasty, Coronary Coronary Balloon Angioplasties Coronary Balloon Angioplasty Coronary Angioplasty, Transluminal Balloon Balloon Dilation, Coronary Artery Transluminal Coronary Balloon Dilation Angioplasty, Transluminal, Percutaneous Coronary Percutaneous Transluminal Coronary Angioplasty Coronary Intervention, Percutaneous Coronary Interventions, Percutaneous Intervention, Percutaneous Coronary Interventions, Percutaneous Coronary Percutaneous Coronary Interventions Percutaneous Coronary Revascularization Coronary Revascularization, Percutaneous Coronary Revascularizations, Percutaneous Percutaneous Coronary Revascularizations Revascularization, Percutaneous Coronary Revascularizations, Percutaneous Coronary Acute Coronary Syndromes Coronary Syndrome, Acute Coronary Syndromes, Acute Syndrome, Acute Coronary Syndromes, Acute Coronary	Hydroxymethylglutaryl-CoA Reductase Inhibitors Hydroxymethylglutaryl CoA Reductase Inhibitors Inhibitors, Hydroxymethylglutaryl-CoA Reductase Reductase Inhibitors, Hydroxymethylglutaryl-CoA HMG-CoA Reductase Inhibitor HMG CoA Reductase Inhibitor Statin Statins Inhibitors, HMG-CoA Reductase Inhibitors, HMG CoA Reductase Reductase Inhibitors, HMG-CoA HMG-CoA Reductase Inhibitors HMG CoA Reductase Inhibitors Inhibitors, Hydroxymethylglutaryl-Coenzyme A Hydroxymethylglutaryl-Coenzyme A Inhibitors Inhibitors, Hydroxymethylglutaryl Coenzyme A Inhibitors, Hydroxymethylglutaryl-CoA Hydroxymethylglutaryl-CoA Inhibitors Inhibitors, Hydroxymethylglutaryl CoA Hydroxymethylglutaryl-CoA Reductase Inhibitor Hydroxymethylglutaryl CoA Reductase Inhibitor Reductase Inhibitor, Hydroxymethylglutaryl-CoA Statins, HMG-CoA HMG-CoA Statins Statins, HMG CoA Simvastatin Atorvastatin Rosuvastatin Calcium Pravastatin	Contrast-induced nephropathy Acute Kidney Injuries Kidney Injuries, Acute Kidney Injury, Acute Acute Renal Injury Acute Renal Injuries Renal Injuries, Acute Renal Injury, Acute Kidney Failure, Acute Acute Kidney Failures Kidney Failures, Acute Acute Kidney Failure Acute Renal Failure Acute Renal Failures Renal Failures, Acute Renal Failure, Acute Renal Insufficiency, Acute Acute Renal Insufficiencies Renal Insufficiencies, Acute Acute Kidney Insufficiency Acute Renal Insufficiency Kidney Insufficiency, Acute Acute Kidney Insufficiencies Kidney Insufficiencies, Acute

 The search strategy used is presented in Table 3.

**Table 3:** Search Strategy

Database	Search strategy	Articles retrieved
PubMed	((((((((((((((((((((((((Acute Kidney Injuries) OR (Kidney Injuries, Acute)) OR (Kidney Injury, Acute)) OR (Acute Renal Injury)) OR (Acute Renal Injuries)) OR (Renal Injuries, Acute)) OR (Renal Injury, Acute)) OR (Kidney Failure, Acute)) OR (Acute Kidney Failures)) OR (Kidney Failures, Acute)) OR (Acute Kidney Failure)) OR (Acute Renal Failure)) OR (Acute Renal Failures)) OR (Renal Failures, Acute)) OR (Renal Failure, Acute)) OR (Renal Insufficiency, Acute)) OR (Acute Renal Insufficiencies)) OR (Renal Insufficiencies, Acute)) OR (Acute Kidney Insufficiency)) OR (Acute Renal Insufficiency)) OR (Kidney Insufficiency, Acute)) OR (Acute Kidney Insufficiencies)) OR (Kidney Insufficiencies, Acute)) AND (((((((((((((((((((((((((((((Hydroxymethylglutaryl-CoA Reductase Inhibitors) OR (Hydroxymethylglutaryl CoA Reductase Inhibitors)) OR (Inhibitors, Hydroxymethylglutaryl-CoA Reductase)) OR (Reductase Inhibitors, Hydroxymethylglutaryl-CoA)) OR (HMG-CoA Reductase Inhibitor)) OR (HMG CoA Reductase Inhibitor)) OR (Statin)) OR (Statins)) OR (Inhibitors, HMG-CoA Reductase)) OR (Inhibitors, HMG CoA Reductase)) OR (Reductase Inhibitors, HMG-CoA)) OR (HMG-CoA Reductase Inhibitors)) OR (HMG CoA Reductase Inhibitors)) OR (Inhibitors, Hydroxymethylglutaryl-Coenzyme A)) OR (Hydroxymethylglutaryl-Coenzyme A Inhibitors)) OR (Inhibitors, Hydroxymethylglutaryl Coenzyme A)) OR (Inhibitors, Hydroxymethylglutaryl-CoA)) OR (Hydroxymethylglutaryl-CoA Inhibitors)) OR (Inhibitors, Hydroxymethylglutaryl CoA)) OR (Hydroxymethylglutaryl-CoA Reductase Inhibitor)) OR (Hydroxymethylglutaryl CoA Reductase Inhibitor)) OR (Reductase Inhibitor, Hydroxymethylglutaryl-CoA)) OR (Statins, HMG-CoA)) OR (HMG-CoA Statins)) OR (Statins, HMG CoA)) OR (Simvastatin)) OR (Atorvastatin)) OR (Rosuvastatin)) OR (Pravastatin))) AND (((((((((((((((((((((((((((((((Coronary angiography) OR (Angiography, Coronary)) OR (Angiographies, Coronary)) OR (Coronary Angiographies)) OR (Angioplasty, Coronary Balloon)) OR (Angioplasties, Coronary Balloon)) OR (Balloon Angioplasties, Coronary)) OR (Balloon Angioplasty, Coronary)) OR (Coronary Balloon Angioplasties)) OR (Coronary Balloon Angioplasty)) OR (Coronary Angioplasty, Transluminal Balloon)) OR (Balloon Dilation, Coronary Artery)) OR (Transluminal Coronary Balloon Dilation)) OR (Angioplasty, Transluminal, Percutaneous)) OR (Percutaneous Transluminal Coronary Angioplasty)) OR (Coronary Intervention, Percutaneous)) OR (Coronary Interventions, Percutaneous)) OR (Intervention, Percutaneous Coronary)) OR (Interventions, Percutaneous Coronary)) OR (Percutaneous Coronary Interventions)) OR (Percutaneous Coronary Revascularization)) OR (Coronary Revascularization, Percutaneous)) OR (Coronary Revascularizations, Percutaneous)) OR (Percutaneous Coronary Revascularizations)) OR (Revascularization, Percutaneous Coronary)) OR (Revascularizations, Percutaneous Coronary)) OR (Acute Coronary Syndromes)) OR (Coronary Syndrome, Acute)) OR (Coronary Syndromes, Acute)) OR (Syndrome, Acute Coronary)) OR (Syndromes, Acute Coronary)) (filters: from 2009 to 2024)	239
Embase	('coronary angiography'/exp OR 'angiography, coronary' OR 'arteriography, coronary' OR 'coronarography' OR 'coronary angiography' OR 'coronary arteriogram' OR 'coronary arteriography' OR 'coronary arteriograpy' OR 'acute coronary syndrome'/exp OR 'acute coronary syndrome' OR 'acute coronary syndromes' OR 'percutaneous coronary intervention'/exp OR 'percutaneous coronary intervention') AND ('statin (protein)'/exp OR 'cell nucleus protein statin' OR 'statin' OR 'statin (protein)' OR 'hydroxymethylglutaryl coenzyme a reductase inhibitor'/exp OR 'atorvastatin'/exp OR '2 (4 fluorophenyl) beta, delta dihydroxy 5 (2 propanyl) 3 phenyl 4 phenylaminocarbonyl 1h pyrrole 1 heptanoic acid' OR '2 (4 fluorophenyl) beta, delta dihydroxy 5 (2 propanyl) 3 phenyl 4 phenylcarbamoyl 1h pyrrole 1 heptanoic acid' OR '2 (4 fluorophenyl) beta, delta dihydroxy 5 isopropyl 3 phenyl 4 phenylaminocarbonyl 1h pyrrole 1 heptanoic acid' OR '2 (4 fluorophenyl) beta, delta dihydroxy 5 isopropyl 3 phenyl 4 phenylcarbamoyl 1h pyrrole 1 heptanoic acid' OR '2 (4 fluorophenyl) beta, delta dihydroxy 5 propan 2 yl 3 phenyl 4 phenylaminocarbonyl 1h pyrrole 1 heptanoic acid' OR '2 (4 fluorophenyl) beta, delta dihydroxy 5 propan 2 yl 3 phenyl 4 phenylcarbamoyl 1h pyrrole 1 heptanoic acid' OR '7 [2 (4 fluorophenyl) 3 phenyl 4 (phenylaminocarbonyl) 5 (2 propanyl) 1 pyrrolyl] 3, 5 dihydroxyheptanoic acid' OR '7 [2 (4 fluorophenyl) 3 phenyl 4 (phenylaminocarbonyl) 5 isopropyl 1 pyrrolyl] 3, 5 dihydroxyheptanoic acid' OR '7 [2 (4 fluorophenyl) 3 phenyl 4 (phenylaminocarbonyl) 5 isopropylpyrrol 1 yl] 3, 5 dihydroxyheptanoic acid' OR '7 [2 (4 fluorophenyl) 3 phenyl 4 (phenylaminocarbonyl) 5 propan 2 ylpyrrol 1 yl] 3, 5 dihydroxyheptanoic acid' OR '7 [2 (4 fluorophenyl) 3 phenyl 4 (phenylcarbamoyl) 5 (2 propanyl) 1 pyrrolyl] 3, 5 dihydroxyheptanoic acid' OR '7 [2 (4 fluorophenyl) 3 phenyl 4 (phenylcarbamoyl) 5 isopropyl 1 pyrrolyl] 3, 5 dihydroxyheptanoic acid' OR '7 [2 (4 fluorophenyl) 3 phenyl 4 (phenylcarbamoyl) 5 isopropylpyrrol 1 yl] 3, 5 dihydroxyheptanoic acid' OR '7 [2 (4 fluorophenyl) 3 phenyl 4 (phenylcarbamoyl) 5 propan 2 ylpyrrol 1 yl] 3, 5 dihydroxyheptanoic acid' OR 'a 2581175' OR 'a2581175' OR 'amicor' OR 'antorcin' OR 'apo-atorva' OR 'arkas' OR 'artas' OR 'ascord' OR 'astator' OR 'atacor' OR 'atofast' OR 'ator' OR 'ator-chol' OR 'atorab' OR 'atorbir' OR 'atorcor' OR 'atoridor' OR 'atorin' OR 'atoris' OR 'atorlip' OR 'atormax' OR 'atorstad' OR 'atorstan' OR 'atorstat' OR 'atorvadivid' OR 'atorvagen' OR 'atorval' OR 'atorvalan' OR 'atorvalet' OR 'atorvaliq' OR 'atorvanox' OR 'atorvastatin' OR 'atorvastatin calcium' OR 'atorvastatin calcium trihydrate' OR 'atorvasterol' OR 'atorvastine' OR 'atorvox' OR 'atorzem' OR 'atostat' OR 'atostin' OR 'atovans' OR 'atovarol' OR 'atractin' OR 'atraven' OR 'atrocell' OR 'atrost' OR 'atrox' OR 'calipra' OR 'card-ok' OR 'cardiostyl' OR 'cardyl' OR 'ci 981' OR 'ci981' OR 'decholest' OR 'delipost' OR 'gletor' OR 'glustar' OR 'lambrinex' OR 'larus' OR 'latrovin' OR 'lipibec' OR 'lipigan' OR 'lipimed' OR 'lipistad' OR 'lipitor' OR 'lipovast' OR 'liprimar' OR 'liptin' OR 'liptonorm' OR 'lorvaten' OR 'lowlipen' OR 'nelibat' OR 'obradon' OR 'omegastatin' OR 'orbeos' OR 'orvasta' OR 'pd 13429838a' OR 'pd13429838a' OR 'prevencor' OR 'provicard' OR 'rafitin' OR 'rotacor' OR 'rotova' OR 's 05153' OR 's05153' OR 'sortis' OR 'statorva' OR 'storvas' OR 'tahor' OR 'thervan' OR 'torvacard' OR 'torvacard neo' OR 'torvacard novum' OR 'torvachol' OR 'torvacol' OR 'torvalipin' OR 'torvaplus' OR 'torvarin' OR 'torvas' OR 'torvast' OR 'torvastin' OR 'torvatec' OR 'torvazin' OR 'totalip' OR 'tulip (drug)' OR 'vastat' OR 'vastatin (atorvastatin)' OR 'vastazor' OR 'voredanin' OR 'xarator' OR 'xolister-all' OR 'ym 548' OR 'ym548' OR 'zarastin' OR 'zarator' OR 'rosuvastatin'/exp OR '7 [4 (4 fluorophenyl) 6 isopropyl 2 (n methylmethanesulfonamido) 5 pyrimidinyl] 3, 5 dihydroxy 6 heptenoic acid' OR '7 [4 (4 fluorophenyl) 6 isopropyl 2 (n methylmethanesulfonamido) pyrimidin 5 yl] 3, 5 dihydroxyhept 6 enoic acid' OR 'coupet' OR 'crestor' OR 'epri' OR 'ezallor' OR 'ezallor sprinkle' OR 'hgp 0816' OR 'hgp0816' OR 'mertenil' OR 'provisacor' OR 'ropitor' OR 'rostat' OR 'rosudia' OR 'rosumop' OR 'rosuvador' OR 'rosuvas' OR 'rosuvastatin' OR 'rosuvastatin calcium' OR 'rosuvastatina' OR 'rosuvastatine' OR 'roswera' OR 'roxera' OR 'rozuva-teva' OR 's 4522' OR 's4522' OR 'simestat' OR 'sorvasta' OR 'visacor (rosuvastatin)' OR 'xeter' OR 'zahron' OR 'zaranta' OR 'zd 4522' OR 'zd4522' OR 'simvastatin'/exp OR '8 [2 (4 hydroxy 6 oxotetrahydro 2h pyran 2 yl) ethyl] 1, 2, 3, 7, 8, 8a hexahydro 3, 7 dimethyl 1 naphthalenyl 2, 2 dimethylbutanoate' OR '8 [2 (4 hydroxy 6 oxotetrahydro 2h pyran 2 yl) ethyl] 1, 2, 3, 7, 8, 8a hexahydro 3, 7 dimethyl 1 naphthyl 2, 2 dimethylbutanoate' OR '8 [2 (4 hydroxy 6 oxotetrahydro 2h pyran 2 yl) ethyl] 3, 7 dimethyl 1, 2, 3, 7, 8, 8a hexahydro 1 naphthalenyl 2, 2 dimethylbutanoate' OR '8 [2 (4 hydroxy 6 oxotetrahydro 2h pyran 2 yl) ethyl] 3, 7 dimethyl 1, 2, 3, 7, 8, 8a hexahydro 1 naphthyl 2, 2 dimethylbutanoate' OR '8 [2 (4 hydroxy 6 oxotetrahydro 2h pyran 2 yl) ethyl] 3, 7 dimethyl 1, 2, 3, 7, 8, 8a hexahydronaphth 1 yl 2, 2 dimethylbutanoate' OR '8 [2 (4 hydroxy 6 oxotetrahydro 2h pyran 2 yl) ethyl] 3, 7 dimethyl 1, 2, 3, 7, 8, 8a hexahydronaphthalen 1 yl 2, 2 dimethylbutanoate' OR 'alcosin' OR 'antichol' OR 'apo-simva' OR 'avastinee' OR 'belmalip' OR 'cholemed' OR 'cholestat' OR 'clinfar' OR 'colastatina' OR 'coledis' OR 'colemin' OR 'colemin forte' OR 'colestricon' OR 'corolin' OR 'corsim' OR 'covastin' OR 'denan' OR 'epistatin' OR 'esvat' OR 'ethicol' OR 'eucor' OR 'flolipid' OR 'glipal' OR 'ifistatin' OR 'ipramid' OR 'jabastatina' OR 'kavelor' OR 'klonastin' OR 'kolestevan' OR 'kymazol' OR 'l 644128' OR 'l644128' OR 'labistatin' OR 'lepur' OR 'lip-down' OR 'lipaxin (simvastatin)' OR 'lipcut' OR 'lipecor' OR 'lipex' OR 'lipinorm' OR 'lipomin' OR 'liponorm' OR 'liporex' OR 'lipovas' OR 'lipterra' OR 'lodales' OR 'lowcholid' OR 'medipo' OR 'medistatin' OR 'mersivas' OR 'mk 0733' OR 'mk 733' OR 'mk0733' OR 'mk733' OR 'nezatin' OR 'nitastin' OR 'nivelipol' OR 'nor-vastina' OR 'normofat' OR 'nyzoc' OR 'omistat' OR 'orovas' OR 'pantok' OR 'pantok forte' OR 'placol' OR 'pravostin' OR 'priacin' OR 'protecta (drug)' OR 'ranzolont' OR 'raptor (drug)' OR 'rechol' OR 'redusterol' OR 'rendapid' OR 'simar' OR 'simbado' OR 'simbatrix' OR 'simcard' OR 'simchol' OR 'simcovas' OR 'simorion' OR 'simovil' OR 'simratio' OR 'simtan' OR 'simtin' OR 'simva' OR 'simvabeta' OR 'simvacard' OR 'simvachol' OR 'simvacol' OR 'simvacor' OR 'simvador' OR 'simvadura' OR 'simvagen' OR 'simvahex' OR 'simvahexal' OR 'simvalid' OR 'simvalimit' OR 'simvalord' OR 'simvasim' OR 'simvastar' OR 'simvastatin' OR 'simvastatina' OR 'simvastatine' OR 'simvasterol' OR 'simvata' OR 'simvatin' OR 'simvax' OR 'simvor' OR 'simvotin' OR 'sincol' OR 'sinpor' OR 'sintenal' OR 'sinvacor' OR 'sinvalip' OR 'sinvastatin' OR 'sinvinolin' OR 'sivastin' OR 'sivatin' OR 'starzoco' OR 'stasiva' OR 'statinum' OR 'stativer' OR 'synvinolin' OR 'torio' OR 'tremital' OR 'valemia' OR 'vasilip' OR 'vasotenal' OR 'vastan' OR 'vastatin (simvastatin)' OR 'vastin (simvastatin)' OR 'vazim' OR 'velastatin' OR 'velkastatin' OR 'velostatin' OR 'veristin' OR 'veritrat' OR 'vidastat' OR 'ximve' OR 'xipocol' OR 'zeplan' OR 'zimmex' OR 'zocor' OR 'zocor forte' OR 'zocord' OR 'zorced' OR 'zosta' OR 'zovar' OR 'zovast' OR 'pravastatin'/exp OR '1, 2, 6, 7, 8, 8a hexahydro beta, delta, 6 trihydroxy 2 methyl 8 (2 methyl 1 oxobutoxy) 1 naphthaleneheptanoic acid' OR '1, 2, 6, 7, 8, 8a hexahydro beta, delta, 6 trihydroxy 2 methyl 8 (2 methyl 1 oxobutoxy) 1 naphthalenylheptanoic acid' OR '1, 2, 6, 7, 8, 8a hexahydro beta, delta, 6 trihydroxy 2 methyl 8 (2 methyl 1 oxobutoxy) 1 naphthylheptanoic acid' OR '1, 2, 6, 7, 8, 8a hexahydro beta, delta, 6 trihydroxy 2 methyl 8 (2 methyl 1 oxobutoxy) naphth 1 ylheptanoic acid' OR '1, 2, 6, 7, 8, 8a hexahydro beta, delta, 6 trihydroxy 2 methyl 8 (2 methyl 1 oxobutoxy) naphthalen 1 ylheptanoic acid' OR '3, 5 dihydroxy 7 [1, 2, 6, 7, 8, 8a hexahydro 6 hydroxy 2 methyl 8 (2 methylbutanoyloxy) 1 naphthalenyl] heptanoic acid' OR '3, 5 dihydroxy 7 [1, 2, 6, 7, 8, 8a hexahydro 6 hydroxy 2 methyl 8 (2 methylbutanoyloxy) 1 naphthyl] heptanoic acid' OR '3, 5 dihydroxy 7 [1, 2, 6, 7, 8, 8a hexahydro 6 hydroxy 2 methyl 8 (2 methylbutanoyloxy) naphth 1 yl] heptanoic acid' OR '3, 5 dihydroxy 7 [1, 2, 6, 7, 8, 8a hexahydro 6 hydroxy 2 methyl 8 (2 methylbutanoyloxy) naphthalen 1 yl] heptanoic acid' OR '3, 5 dihydroxy 7 [1, 2, 6, 7, 8, 8a hexahydro 6 hydroxy 2 methyl 8 (2 methylbutyryloxy) 1 naphthalenyl] heptanoic acid' OR '3, 5 dihydroxy 7 [1, 2, 6, 7, 8, 8a hexahydro 6 hydroxy 2 methyl 8 (2 methylbutyryloxy) 1 naphthyl] heptanoic acid' OR '3, 5 dihydroxy 7 [1, 2, 6, 7, 8, 8a hexahydro 6 hydroxy 2 methyl 8 (2 methylbutyryloxy) naphth 1 yl] heptanoic acid' OR '3, 5 dihydroxy 7 [1, 2, 6, 7, 8, 8a hexahydro 6 hydroxy 2 methyl 8 (2 methylbutyryloxy) naphthalen 1 yl] heptanoic acid' OR '3, 5 dihydroxy 7 [6 hydroxy 2 methyl 8 [ (2 methylbutanoyl) oxy] 1, 2, 6, 7, 8, 8a hexahydro 1 naphthalenyl] heptanoic acid' OR '3, 5 dihydroxy 7 [6 hydroxy 2 methyl 8 [ (2 methylbutanoyl) oxy] 1, 2, 6, 7, 8, 8a hexahydro 1 naphthyl] heptanoic acid' OR '3, 5 dihydroxy 7 [6 hydroxy 2 methyl 8 [ (2 methylbutanoyl) oxy] 1, 2, 6, 7, 8, 8a hexahydronaphth 1 yl] heptanoic acid' OR '3, 5 dihydroxy 7 [6 hydroxy 2 methyl 8 [ (2 methylbutanoyl) oxy] 1, 2, 6, 7, 8, 8a hexahydronaphthalen 1 yl] heptanoic acid' OR '3, 5 dihydroxy 7 [6 hydroxy 2 methyl 8 [ (2 methylbutyryl) oxy] 1, 2, 6, 7, 8, 8a hexahydro 1 naphthalenyl] heptanoic acid' OR '3, 5 dihydroxy 7 [6 hydroxy 2 methyl 8 [ (2 methylbutyryl) oxy] 1, 2, 6, 7, 8, 8a hexahydro 1 naphthyl] heptanoic acid' OR '3, 5 dihydroxy 7 [6 hydroxy 2 methyl 8 [ (2 methylbutyryl) oxy] 1, 2, 6, 7, 8, 8a hexahydronaphth 1 yl] heptanoic acid' OR '3, 5 dihydroxy 7 [6 hydroxy 2 methyl 8 [ (2 methylbutyryl) oxy] 1, 2, 6, 7, 8, 8a hexahydronaphthalen 1 yl] heptanoic acid' OR '7 [1, 2, 6, 7, 8, 8a hexahydro 6 hydroxy 2 methyl 8 (2 methylbutanoyloxy) 1 naphthalenyl] 3, 5 dihydroxyheptanoic acid' OR '7 [1, 2, 6, 7, 8, 8a hexahydro 6 hydroxy 2 methyl 8 (2 methylbutanoyloxy) 1 naphthyl] 3, 5 dihydroxyheptanoic acid' OR '7 [1, 2, 6, 7, 8, 8a hexahydro 6 hydroxy 2 methyl 8 (2 methylbutanoyloxy) naphth 1 yl] 3, 5 dihydroxyheptanoic acid' OR '7 [1, 2, 6, 7, 8, 8a hexahydro 6 hydroxy 2 methyl 8 (2 methylbutanoyloxy) naphthalen 1 yl] 3, 5 dihydroxyheptanoic acid' OR '7 [1, 2, 6, 7, 8, 8a hexahydro 6 hydroxy 2 methyl 8 (2 methylbutyryloxy) 1 naphthalenyl] 3, 5 dihydroxyheptanoic acid' OR '7 [1, 2, 6, 7, 8, 8a hexahydro 6 hydroxy 2 methyl 8 (2 methylbutyryloxy) 1 naphthyl] 3, 5 dihydroxyheptanoic acid' OR '7 [1, 2, 6, 7, 8, 8a hexahydro 6 hydroxy 2 methyl 8 (2 methylbutyryloxy) naphth 1 yl] 3, 5 dihydroxyheptanoic acid' OR '7 [1, 2, 6, 7, 8, 8a hexahydro 6 hydroxy 2 methyl 8 (2 methylbutyryloxy) naphthalen 1 yl] 3, 5 dihydroxyheptanoic acid' OR '7 [6 hydroxy 2 methyl 8 (2 methylbutanoyl) oxy 1, 2, 6, 7, 8, 8a hexahydronaphthalen 1 yl] 3, 5 dihydroxyheptanoic acid' OR '7 [6 hydroxy 2 methyl 8 [ (2 methylbutanoyl) oxy] 1, 2, 6, 7, 8, 8a hexahydro 1 naphthalenyl] 3, 5 dihydroxyheptanoic acid' OR '7 [6 hydroxy 2 methyl 8 [ (2 methylbutanoyl) oxy] 1, 2, 6, 7, 8, 8a hexahydro 1 naphthyl] 3, 5 dihydroxyheptanoic acid' OR '7 [6 hydroxy 2 methyl 8 [ (2 methylbutanoyl) oxy] 1, 2, 6, 7, 8, 8a hexahydronaphth 1 yl] 3, 5 dihydroxyheptanoic acid' OR '7 [6 hydroxy 2 methyl 8 [ (2 methylbutyryl) oxy] 1, 2, 6, 7, 8, 8a hexahydro 1 naphthalenyl] 3, 5 dihydroxyheptanoic acid' OR '7 [6 hydroxy 2 methyl 8 [ (2 methylbutyryl) oxy] 1, 2, 6, 7, 8, 8a hexahydro 1 naphthyl] 3, 5 dihydroxyheptanoic acid' OR '7 [6 hydroxy 2 methyl 8 [ (2 methylbutyryl) oxy] 1, 2, 6, 7, 8, 8a hexahydronaphth 1 yl] 3, 5 dihydroxyheptanoic acid' OR '7 [6 hydroxy 2 methyl 8 [ (2 methylbutyryl) oxy] 1, 2, 6, 7, 8, 8a hexahydronaphthalen 1 yl] 3, 5 dihydroxyheptanoic acid' OR 'aplactin' OR 'astin' OR 'bristacol' OR 'cholespar' OR 'cs 514' OR 'cs514' OR 'dehypotin protect' OR 'elisor' OR 'epatostantin' OR 'eptastatin' OR 'eptastatin sodium' OR 'eptastatine' OR 'kenstatin' OR 'lipemol' OR 'lipidal' OR 'liplat' OR 'lipostat' OR 'liprevil' OR 'maxudin' OR 'mevalotin' OR 'minuscol' OR 'novales' OR 'prareduct' OR 'prascolend' OR 'prastan' OR 'prasterol' OR 'prava' OR 'pravachol' OR 'pravacol' OR 'pravalam' OR 'pravalich' OR 'pravaselect' OR 'pravasin' OR 'pravasine' OR 'pravastatin' OR 'pravastatin natrium mayrho fer' OR 'pravastatin sodium' OR 'pravastatine' OR 'pravator' OR 'pravyl' OR 'sanaprav' OR 'selectin (drug)' OR 'selektine' OR 'selipran' OR 'sq 31000' OR 'sq31000' OR 'stanidine' OR 'vasopran' OR 'vasten' OR 'versatab' OR 'xipral') AND ('acute kidney failure'/exp OR 'acute kidney failure' OR 'acute kidney injury' OR 'acute kidney insufficiency' OR 'acute renal failure' OR 'acute renal insufficiency' OR 'kidney acute failure' OR 'kidney failure, acute' OR 'kidney insufficiency, acute' OR 'renal insufficiency, acute' OR 'contrast induced nephropathy'/exp OR 'rc-induced nephropathy' OR 'contrast agent induced nephropathy' OR 'contrast agent induced nephrotoxicity' OR 'contrast induced acute renal failure' OR 'contrast induced nephropathy' OR 'contrast induced nephrotoxicity' OR 'contrast induced renal dysfunction' OR 'contrast induced renal failure' OR 'contrast media induced nephropathy' OR 'contrast media induced nephrotoxicity' OR 'contrast media induced renal failure' OR 'contrast medium induced nephropathy' OR 'contrast medium induced nephrotoxicity' OR 'contrast medium induced renal failure' OR 'contrast nephropathy' OR 'contrast nephrotoxicity' OR 'contrasting agent-induced nephropathy' OR 'radio-contrast nephropathy' OR 'radio-contrast-induced nephropathy' OR 'radiocontrast nephropathy' OR 'radiocontrast-induced nephropathy') AND (2009-2024)/py	850

 Table 4 and Table 5 show the baseline characteristics of the included studies.

**Table 4:** Baseline Characteristics of the Included Studies HDS: high-dose statins, BMI: body mass index, HTN: hypertension, CKD: chronic kidney disease

Study	Mean age		Male (number)		Mean BMI		HTN (number)		CKD (number)	
	HDS	Control	HDS	Control	HDS	Control	HDS	Control	HDS	Control
Leoncini et al. (2014) [17]	66.2	66.1	166	165	26.2	26.6	143	138	39	37
Abaci et al. (2015) [18]	67.5	67.7	66	76	-	-	91	92	103	105
de Oliveira et al. (2012) [19]	59.4	62.2	38	52	-	-	-	-	8	10
Bidram et al. (2015) [20]	59.9	60.4	89	92	26.7	26.83	-	-	-	-
Patti et al. (2011) [21]	65	66	91	96	26.9	26.8	91	90	35	39
Quintavalle et al. (2012) [22]	70	70	103	120	28	28	172	182	202	208
Syed et al. (2017) [23]	53.1	51.8	47	52	-	-	80	80	-	-
Khosravi et al. (2016) [24]	-	-	-	-	-	-	-	-	110	110
Shehata et al. (2015) [25]	55	57	35	32	27	28	34	35	65	65
Hammami et al. (2023) [13]	60.8	62.2	38	41	27.6	25.8	35	32	17	22
Watanabe et al. (2022) [26]	73.6	73.7	150	149	24.5	24.6	164	160	203	203

**Table 5:** Baseline Characteristics of the Included Studies HDS: high-dose statins, DM: diabetes mellitus

Study	DM (number)		Dyslipidemia (number)		Smoking (number)		Mean baseline serum creatinine		Mean/median volume of contrast (mL)	
	HDS	Control	HDS	Control	HDS	Control	HDS	Control	HDS	Control
Leoncini et al. (2014) [17]	50	57	-	-	89	81	0.95	0.96	149.7	138.2
Abaci et al. (2015) [18]	53	50	42	37	31	27	1.3	1.4	139.2	117.7
de Oliveira et al. (2012) [19]	20	22	49	53	14	17	0.89	0.98	72.2	78.2
Bidram et al. (2015) [20]	-	-	-	-	-	-	1.18	1.14	-	-
Patti et al. (2011) [21]	36	32	31	33	39	29	01.04	01.04	209	213
Quintavalle et al. (2012) [22]	89	80	-	-	-	-	1.32	1.29	177	184
Syed et al. (2017) [23]	80	80	-	-	-	-	1.12	1.15	-	-
Khosravi et al. (2016) [24]	110	110	-	-	-	-	1.53	1.47	-	-
Shehata et al. (2015) [25]	65	65	29	30	27	26	2	2	274	278
Hammami et al. (2023) [13]	32	23	42	48	16	16	0.75	0.80	60	60
Watanabe et al. (2022) [26]	102	85	-	-	123	127	1.21	1.19	90	82

 The Cochrane Risk of Bias (RoB) tool assessment for the included randomized controlled trials (RCTs) is shown in Figure 4.

## References

[1] Lasser EC, Lyon SG, Berry CC (1997). Reports on contrast media reactions: analysis of data from reports to the U.S. Food and Drug Administration. Radiology.

[2] Mehran R, Nikolsky E (2006). Contrast-induced nephropathy: definition, epidemiology, and patients at risk. Kidney Int Suppl.

[3] Tsai TT, Patel UD, Chang TI (2014). Contemporary incidence, predictors, and outcomes of acute kidney injury in patients undergoing percutaneous coronary interventions: insights from the NCDR Cath-PCI registry. JACC Cardiovasc Interv.

[4] Ni Z, Liang Y, Xie N (2019). Simple pre-procedure risk stratification tool for contrast-induced nephropathy. J Thorac Dis.

[5] Li Y, Wang J (2024). Contrast-induced acute kidney injury: a review of definition, pathogenesis, risk factors, prevention and treatment. BMC Nephrol.

[6] Oesterle A, Laufs U, Liao JK (2017). Pleiotropic effects of statins on the cardiovascular system. Circ Res.

[7] Koushki K, Shahbaz SK, Mashayekhi K (2021). Anti-inflammatory action of statins in cardiovascular disease: the role of inflammasome and Toll-like receptor pathways. Clin Rev Allergy Immunol.

[8] Leoncini M, Toso A, Maioli M, Tropeano F, Bellandi F (2013). Statin treatment before percutaneous cononary intervention. J Thorac Dis.

[9] Zhou YL, Chen LQ, Du XG (2021). Efficacy of short-term moderate or high-dose statin therapy for the prevention of contrast-induced nephropathy in high-risk patients with chronic kidney disease: systematic review and meta-analysis. Clinics (Sao Paulo).

[10] Dashti-Khavidaki S, Moghaddas A, Heydari B, Khalili H, Lessan-Pezeshki M, Lessan-Pezeshki M (2013). Statins against drug-induced nephrotoxicity. J Pharm Pharm Sci.

[11] Zhang L, Zhang L, Lu Y (2011). Efficacy of statin pretreatment for the prevention of contrast-induced nephropathy: a meta-analysis of randomised controlled trials. Int J Clin Pract.

[12] Thompson K, Razi R, Lee MS (2016). Statin use prior to angiography for the prevention of contrast-induced acute kidney injury: a meta-analysis of 19 randomised trials. EuroIntervention.

[13] Hammami R, Masmoudi O, Jdidi J (2023). Impact of atorvastatin reload on the prevention of contrast-induced nephropathy in patients on chronic statin therapy: a prospective randomized trial. PLoS One.

[14] Hariton E, Locascio JJ (2018). Randomised controlled trials - the gold standard for effectiveness research: study design: randomised controlled trials. BJOG.

[15] Page MJ, McKenzie JE, Bossuyt PM (2021). The PRISMA 2020 statement: an updated guideline for reporting systematic reviews. BMJ.

[16] Cumpston M, Li T, Page MJ, Chandler J, Welch VA, Higgins JP, Thomas J (2019). Updated guidance for trusted systematic reviews: a new edition of the Cochrane Handbook for Systematic Reviews of Interventions. Cochrane Database Syst Rev.

[17] Leoncini M, Toso A, Maioli M, Tropeano F, Villani S, Bellandi F (2014). Early high-dose rosuvastatin for contrast-induced nephropathy prevention in acute coronary syndrome: results from the PRATO-ACS Study (Protective Effect of Rosuvastatin and Antiplatelet Therapy On contrast-induced acute kidney injury and myocardial damage in patients with Acute Coronary Syndrome). J Am Coll Cardiol.

[18] Abaci O, Arat Ozkan A, Kocas C (2015). Impact of rosuvastatin on contrast-induced acute kidney injury in patients at high risk for nephropathy undergoing elective angiography. Am J Cardiol.

[19] de Oliveira MS, Bomfim Araujo Martins K, Ribamar Costa J (2012). Impact on renal function of rosuvastatin preload prior to elective percutaneous coronary intervention in chronic statin users. Rev Bras Cardiol Invasiva (Engl Ed).

[20] Bidram P, Roghani F, Sanei H (2015). Atorvastatin and prevention of contrast induced nephropathy following coronary angiography. J Res Med Sci.

[21] Patti G, Ricottini E, Nusca A (2011). Short-term, high-dose atorvastatin pretreatment to prevent contrast-induced nephropathy in patients with acute coronary syndromes undergoing percutaneous coronary intervention (from the ARMYDA-CIN [atorvastatin for reduction of myocardial damage during angioplasty--contrast-induced nephropathy] trial. Am J Cardiol.

[22] Quintavalle C, Fiore D, De Micco F (2012). Impact of a high loading dose of atorvastatin on contrast-induced acute kidney injury. Circulation.

[23] Syed MH, Khandelwal PN, Thawani VR, Katare SS (2017). Efficacy of atorvastatin in prevention of contrast-induced nephropathy in high-risk patients undergoing angiography: a double-blind randomized controlled trial. J Pharmacol Pharmacother.

[24] Khosravi A, Dolatkhah M, Hashemi HS, Rostami Z (2016). Preventive effect of atorvastatin (80 mg) on contrast-induced nephropathy after angiography in high-risk patients: double-blind randomized clinical trial. Nephrourol Mon.

[25] Shehata M, Hamza M (2015). Impact of high loading dose of atorvastatin in diabetic patients with renal dysfunction undergoing elective percutaneous coronary intervention: a randomized controlled trial. Cardiovasc Ther.

[26] Watanabe M, Aonuma K, Murohara T (2022). Prevention of contrast-induced nephropathy after cardiovascular catheterization and intervention with high-dose strong statin therapy in Japan - the PREVENT CINC-J Study. Circ J.

[27] Pranata R, Wahyudi DP (2023). Prevention of contrast-induced nephropathy in patients undergoing percutaneous coronary intervention. Curr Cardiol Rev.

[28] Borkowski P, Borkowska N, Mangeshkar S, Adal BH, Singh N (2024). Racial and socioeconomic determinants of cardiovascular health: a comprehensive review. Cureus.

[29] Cheng AS, Li X (2023). The potential biotherapeutic targets of contrast-induced acute kidney injury. Int J Mol Sci.

[30] Ali-Hasan-Al-Saegh S, Mirhosseini SJ, Ghodratipour Z (2017). Strategies preventing contrast-induced nephropathy after coronary angiography: a comprehensive meta-analysis and systematic review of 125 randomized controlled trials. Angiology.

[31] Lin M, Xu T, Zhang W (2021). Effect of statins on post-contrast acute kidney injury: a multicenter retrospective observational study. Lipids Health Dis.

[32] Zhou X, Dai J, Xu X (2019). Comparative efficacy of statins for prevention of contrast-induced acute kidney injury in patients with chronic kidney disease: a network meta-analysis. Angiology.

[33] Cho A, Lee YK, Sohn SY (2020). Beneficial effect of statin on preventing contrast-induced acute kidney injury in patients with renal insufficiency: a meta-analysis. Medicine (Baltimore).

[34] Jasińska M, Owczarek J, Orszulak-Michalak D (2007). Statins: a new insight into their mechanisms of action and consequent pleiotropic effects. Pharmacol Rep.

[35] Nežić L, Škrbić R, Amidžić L (2020). Protective effects of simvastatin on endotoxin-induced acute kidney injury through activation of tubular epithelial cells’ survival and hindering cytochrome c-mediated apoptosis. Int J Mol Sci.

[36] Wong PC, Li Z, Guo J, Zhang A (2012). Pathophysiology of contrast-induced nephropathy. Int J Cardiol.

[37] Li H, Wang C, Liu C, Li R, Zou M, Cheng G (2016). Efficacy of short-term statin treatment for the prevention of contrast-induced acute kidney injury in patients undergoing coronary angiography/percutaneous coronary intervention: a meta-analysis of 21 randomized controlled trials. Am J Cardiovasc Drugs.

[38] Gandhi S, Mosleh W, Abdel-Qadir H, Farkouh ME (2014). Statins and contrast-induced acute kidney injury with coronary angiography. Am J Med.

[39] Acharya T, Huang J, Tringali S, Frei CR, Mortensen EM, Mansi IA (2016). Statin use and the risk of kidney disease with long-term follow-up (8.4-year study). Am J Cardiol.

[40] Chung YH, Lee YC, Chang CH, Lin MS, Lin JW, Lai MS (2013). Statins of high versus low cholesterol-lowering efficacy and the development of severe renal failure. Pharmacoepidemiol Drug Saf.

[41] McMurray JJ, Dunselman P, Wedel H (2010). Coenzyme Q10, rosuvastatin, and clinical outcomes in heart failure: a pre-specified substudy of CORONA (controlled rosuvastatin multinational study in heart failure). J Am Coll Cardiol.

[42] Singh RB, Khanna HK, Niaz MA (2000). Randomized, double-blind placebo-controlled trial of coenzyme Q10 in chronic renal failure: discovery of a new role. J Nutr Environ Med.

[43] Ishikawa A, Kawarazaki H, Ando K, Fujita M, Fujita T, Homma Y (2011). Renal preservation effect of ubiquinol, the reduced form of coenzyme Q10. Clin Exp Nephrol.

[44] Chen F, Liu F, Lu J, Yang X, Xiao B, Jin Y, Zhang J (2018). Coenzyme Q10 combined with trimetazidine in the prevention of contrast-induced nephropathy in patients with coronary heart disease complicated with renal dysfunction undergoing elective cardiac catheterization: a randomized control study and in vivo study. Eur J Med Res.

[45] Zhao S, Wu W, Liao J (2022). Molecular mechanisms underlying the renal protective effects of coenzyme Q10 in acute kidney injury. Cell Mol Biol Lett.

[46] Katsiki N, Fonseca V, Mikhailidis DP (2018). Contrast-induced acute kidney injury in diabetes mellitus: clinical relevance and predisposing factors. Could statins be of benefit?. J Diabetes Complications.

[47] Toso A, Leoncini M, Maioli M, Tropeano F, Di Vincenzo E, Villani S, Bellandi F (2014). Relationship between inflammation and benefits of early high-dose rosuvastatin on contrast-induced nephropathy in patients with acute coronary syndrome: the pathophysiological link in the PRATO-ACS study (Protective Effect of Rosuvastatin and Antiplatelet Therapy on Contrast-Induced Nephropathy and Myocardial Damage in Patients With Acute Coronary Syndrome Undergoing Coronary Intervention). JACC Cardiovasc Interv.

